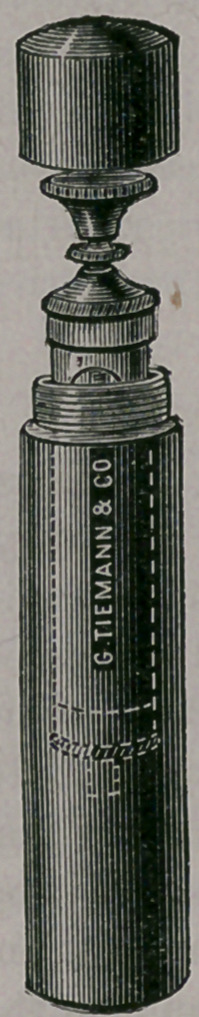# Medical Items and News of Interest to the Physician

**Published:** 1880-07

**Authors:** 


### For the Use of Physicians.

CULLED FROM THE STANDARD MEDICAL JOURNALS OF THE WORLD.

NOTE.—These formulæ are intended only for the regular practitioner of medicine, and should be employed only by him, or under his immediate supervision.

**Cotton-leaf Tea as a Galactagogue.**

Dr. Izett W. Anderson, of Jamaica, reports to the Obstetrical Society of London, that he has found a tea prepared from the green leaves of the *Gossypium barbadense* to be an efficacious galactagogue.

**Painful Hæmorrhoids.**

℞
Extract of henbane,
Powdered saffron, āā ℥ ijss.
Acetatate of lead, ʒ j.
Glycerole of starch, ℥ j.

To be applied twice or three times a day.—*Magaz. of Pharm.*

**Whooping Cough.**

Dr. Pollak, of Austria, recommends the following formula for insufflation in the treatment of whooping cough :

Tannate of quinia,
Bicarbonate of sodium, of each, 5 parts.
Powdered gum arabic, 100 parts.
M.

**Uticaria.**

Bromide of Potassium in solution, one ounce to a pint of water, used externally, will almost invariably allay the irritation of urticaria simplex. This has been the experience of Dr. E. C. Dudley, of Chicago, who has prescribed it frequently and seldom been disappointed in its results. It appears to act as a local sedative to the vaso-motor nerves of the skin.—*Chicago Medical Gazette.*

**Moles or Nævus.**

According to Dr. Sigler, they may be removed by means of croton oil in the following manner. Push a number of needles through a cork, so that the points project 3 to 4 millimetres. Dip the points in croton oil, then insert them in the mole and withdraw. This is a sort of Baunscheidtismus. A scab will form upon the mole ; and after it has dried up and dropped off, the operation is twice more repeated.—*Pharm. Centralh. and Ph. Zeit.*

**To Preserve the Strength of Magendie's Solution.**

The following is the formula of Dr. H. M. Keyes, he having called attention to its successful employment in Roosevelt Hospital, some years ago.

℞
Morph. sulph., gr. cclvj.
Acid. salicylic, gr. viij.
Aq. distil., f. ℥ xvj.

**Whooping Cough.**

A British authority recommends the following as "almost a specific" in this disease :

℞

Quiniæ sulphatis, ℈j.

Sol. acid. hydrobromic (Fothergilll) ʒiss

Syrupi altheæ, ℥iss.

Aquæ, ad ℥vi.

M.

A dessertspoonful four times a day ; the dose to be increased according to age.

**Hydrophobia.**

Dr. Offenberg, in Germany, says the *Apoth Zeit.*, has cured a woman who had been bitten severely by a mad dog, and who exhibited the most unmistakable symptoms of hydrophobia. He injected subcutaneously curare in rather large doses, giving within five hours twenty centigrammes (three grains). The hydrophobia was conquered, but now the specific action of the curare began to manifest itself by paralysis of the heart and of the respiratory organs, which was combated by artificial respiration.

**Puerperal Eclampsia.**

An exclusive Milk Diet as Prophylaxis, is advocated in the Revue de Therapeutique Medico-Chirurgicale. The indication for the treatment is found in the constant presence of albuminuria. The quantity of milk per day to be taken is by direction, to be continued months after the disappearance of the albumen. The average quantity tolerated is three litres per day, persisted in even though there may be great repugnance to the diet. Tarnier cites several successful cases of the kind, and claims that persistence in this regimen invariably caused the pathological condition to disappear, pregnancy terminating without trouble. Gastric derangements arising in the course of this treatment he combated with "ordinary medicines." Measures to prevent constipation or diarrhœa were also taken.—*Chicago Med. Gazette.*

**Diphtheria.**

Dr. Henry Bergeron reports very favorable results from inhalations of an atmosphere containing a definite small proportion of hydrofluoric acid gas. The quantity employed is one gramme (15 grains) for each cubic metre (1-3 cubic yards) of air space in the room during three hours. The gas is generated by allowing the proper amount of acid slowly to evaporate from a leaden vessel placed on a table a short distance from the bed of the patient.

---

**Catgut Ligature.**

Dr. A. T. Cabot exhibited at a recent meeting of the Boston Society for medical improvement an ovarian cyst, removed a week previous by Dr. Homans. There were no adhesions, and the case was favorable for the operation, which lasted forty minutes. The patient died within a few hours. An examination revealed abdominal hemorrhage from the loosening of one of the ligatures. This case shows the danger of the catgut ligature. The experience of the late Dr. Peaslee is corroborative of the above.

---

**Gonorrhœa.**

Dr. H. B. Stanley, of Arbuckle, California, is partial to the following prescription in gonorrhœa :

℞

Fl. ext. cubebs,
Fl. ext. yerba santa,
Balsam copaiba,
Syrup acacia, aa ℥ ss,
Comp. spts. lavender, ʒ ij.

M. Sig. A teaspoonful three times a day. The Spaniards, it is said, treat gonorrhœa by chewing the leaves of the yerba santa.

---

**Transfusion.**

The interest awakened by the successful employment of defibrinated blood, *per rectum*, as a valuable auxiliary in the treatment of disease, leads me to call attention to the experiments of Prof. Ponfick, which have not been recorded, to my knowledge, in any of our journals, and which seem to open a new sphere of usefulness for this agent. I allude to the intraperitoneal injections of defibrinated blood, which Prof. Ponfick ranks as a simple and effective method of transfusion, devoid of the difficulties and dangers attending the ordinary procedure of transferring the blood directly from one person to another.—*Chicago Medical Gazette.*

**Chorea.**

Dr. Mareiglia describes, in an Italian Medical Journal, a translation of which we find in the *Practitioner*, four cases of chorea treated with ether spray, as recommended by Lubelsky. The spray was directed along the entire length of the spine, by means of a Richardson's apparatus, for a breadth of 7 cm. Application was continued from three to four minutes, and made from 2 to 3 times a day. They were all cured in about 50 days. The ether treatment was continued from 17 to 45 days.

---

**Night Sweats.**

We learn from the Practitioner that picrotoxine, the active principle of cocculus indicus, has been used by Dr. William Murrell, M. R. C. P., with great advantage in the treatment of the night sweats of phthisis. The alkaloid is soluable in one hundred and eighty parts of water. As a rule, the doctor says, there is no improvement the first night, but by the third or fourth night the sweating has practically ceased. It returns, however, in from ten to fourteen days, and repetition of the treatment is necessary. The ordinary dose is 1-180 grain, and the greatest freedom of administration recorded is 1-60 grain, four times a day. Heaven's richest blessings upon the man, woman or child who enlarges our resources for arresting night sweats.—*Chicago Med. Gazette.*

---

**Podophyllin.**

All who have been accustomed to prescribe podophyllian in pills will agree as to the impossibility of preventing occasional disastrous effects. But this is the fault of the form of administration, not of the drug. From a very long and extensive experience I can confidently affirm that none of the accidents or inconveniences which so commonly attend the administration of podophollin ever arise when the drug is prescribed according to my method. On the contrary, it is one of the most satisfactory and reliable of our medicines. The formula given is :

℞

Podophilli, gr. ij.
Essentiæ zingiberis, ʒ ij.
Spiritus vini recti q. s. ad. ℥ ij.
Fiat guttæ.

A teaspoonful to be taken in a wineglassful of water every night at bedtime, or every second, third, or fourth night as required.—*Dr. Horace Dobell, British Med. Journal.*

**Diabetes.**

Dr. J. Y. Dale, of Lemont, Pa., writing to the Boston *Medical and Surgical Journal*, says:

It was first brought to my notice in 1876, by a medical friend who had used it with great success. It is the nitrate of uranium, given in doses of one or two grammes three times a day. A year ago I treated a case in which this was the only medicine prescribed, and in connection with appropriate diet the quantity of urine was reduced from two and a half gallons per diem to a quart in less than a month, with corresponding improvement in all the other symptoms.

---

**Fissured Nipples.**

Dr. King, in the St. Louis *Courier of Medicine*, recommends the application to fissured nipples of a solution of gutta percha in benzine or bi-sulphide of carbon. He paints this solution all over the nipple, except the apertures of the milk ducts. It remains on two or three days, and usually the parts are entirely healed. Occasionally it needs to be re-applied. It is suggested that the cement used by cobblers in mending shoes, by what they term "seamless patch," would answer the above indication, but better still, would be to use the officinal solution of gutta percha in chloroform.

---

**Hydrate of Chloral—A Request.**

Physicians everywhere, are earnestly requested to answer the following questions as fully as is possible:

1. How is the taste of chloral best disguised in fluid preparations?

2. Do you believe that physicians are prescribing chloral as extensively to-day as they were five years ago?

3. Does there seem to be any increase in the use of the drug by people, aside from that ordered in prescriptions?

4. Have you any patients who use the drug regularly? If so, please state how often, and in what quantity.

5. Do you know of any cases of death produced by chloral? If so, please state the amount used, the name of the physician who was called to the case, and the name of the coronor, if an inquest was held.

All communications will be considered *strictly confidential*, the writer's name not being used where a request to that effect is made. Address all communications to Dr. H. H. Kane, 266 Bleeker street, New York City.

**Bile in Urine**

The presence of bile in urine is detected, according to Massel, in *Ap. Zeit.*, by adding to about one fluid drachm of urine in a test-tube four or five drops of concentrated sulphuric acid and throwing into it a small crystal of nitrate of potassa. If bile be present in not too small a quantity, emerald green stripes will be seen starting from the edges of the crystal. On shaking, the color of the urine changes to dark green, which does not disappear on boiling. If only mere traces of bilious matter be present, the color changes to a pale green, best noticed by looking from above down iuto the test tube while it is held over a piece of white paper.

---

**Coto Bark in Diarrhœa.**

I have tried coto bark with prompt success in a case of diarrhœa with tubercular complications. The following is the formula I have employed:

℞
Fl. ext. coto bark,
Comp. tr. cardamon, āā ℥ ij.
Mucilage acacia.
Syrup, āā ℥ ss.
Cinnamon water, qs ad ℥ viij.

M. Sig. A tablespoonful every three hours.

—*J. B. Crandall, M. D., Sterling, O.*

---

**Pulsatilla.**

According to Dr. James I. Tucker, in the *Chicago Medical Gazette*, pulsatilla is rapidly growing in favor with many practitioners. Though a very old remedy, having been known to Dioscorides and Pliny, it fell into disuse, if not into disrepute, and was not reinstated till about the beginning of the present century. I have used pulsatilla mainly in simple dysmenorrhœa, and here it has proved of decided utility. Its scope is, however, doubtless much wider. A very prominent lawyer of this city told me, not long since, that after trying the bromides, the valerianates, and other remedies of repute for the headaches caused by excessive mental application, he found no relief till he made use of the tincture of pulsatilla. He is now never without it, and uses no other medicine for the cure of his headache, which I know to be very severe. No such powers are attributed to it in the books to which I have access. This is an exceptional case, it may be, but it is a valid one.

The tincture of pulsatilla should be made from the fresh plant, and given with caution. The dose is from three to ten drops.

**Chest Diseases.**

Dr. Moubre, writing to the *Gazette des Hopitaux*, gives his experience of petroleum capsules in simple and chronic bronchitis. This balsamic had been brought before the Therapeutic Socity by Dr. Blache a year ago, at the request of a Paris chemist, who named it Gabian oil, in order to prevent public prejudice. Each capsule contains twenty-five centigrammes (4 grains) of pure petroleum, the ordinary oil not being used, as it has to be distilled in contact with sulphuric acid to render it fit for lighting purposes. At the Hospital Beaujon, where these capsules have been freely ordered for chronic bronchitis, a rapid diminution of the secretions and fits of coughing were observed. In tuberculosis this medicine gave encouraging results.

---

**Sims' Speculum Always at Hand.**

The index and middle fingers of the right hand may be used as a perineal retractor in place of the ordinary Sims' speculum. They may be introduced with the patient in Sims' latero-prone position, the operator standing back of the patient, on the side of the table, in exactly the position of the assistant, who holds the speculum in the ordinary way. In this manner the cervix and vagina may be exposed almost as well as by the speculum. This method of exposing the parts may be of great use when a speculum is needed and not accessible; in the application, for instance, of the tampon in sudden hemorrhage, or in consultations at a distance, when, for reasons not anticipated, it becomes necessary to examine the pelvic organs.—*Chicago Med. Gazette.*

---

**Cutaneous Affections.**

Dr. John V. Shoemaker strongly recommends the use of oleate of lead as topical application in various skin diseases. The preparation is made by adding liquor potassæ to a diluted preparation of liquor plumbi subacetatis, the precipitate being collected on a filter and dried. The dry suboxide of lead should then be dissolved in oleic acid by means of the water bath. The strength of the solution should be five per cent. of lead to the oleic acid, and as free as possible of steaic and margaric acid, in order to have it in the liquid form. Should either the percentage of lead be increased or the solution contaminated by stearic or margaric acid, the oleate will be semi-solid, and will not have the same efficient action. The oleate of lead is an opaque oily liquid, if prepared with care in the manner indicated. It is a mild astringent, more readily absorbed than either Goulard's cerate or Hebra's litharge ointment; while it possesses the advantage of neither decomposing nor turning rancid. Remarkably good results have been obtained from its use in eczema, in rosalia, after depletion of the parts, in burns, and in erythema. It arrests morbid discharges, protects the surface and allays irritation by its astringent and sedative action.

---

**Alopecia and Psoriasis.**

For alopecia, Dr. John V. Shoemaker says that a ten per cent solution of the oleate of mercury with the addition of a small quantity of olive oil, and scented with some essential oil, is an invaluable application for general thinning and loss of hair. When brushed lightly over the scalp in the above condition it produces both a tonic and alterative effect upon the part. He has also employed as an application with great success a two ounce solution of the oleate of mercury, of ten per cent. strength, mixed with an equal quantity of olive oil, in psoriasis and pityriasis, after all the redness and scales have disappeared. The use of this preparation in these affections protects and soothes the hyperæmic skin, and prevents a return of the diseased condition.

---

**Ringworm.**

A writer in the *British Medical Journal* gives the formula for Coster's paste, thus :

℞

Iodine pigment, ʒij.

Oil of cade or oil of juniper tar, ℥j.

Mix. For an embrocation.

He finds the following formula, however, most effectual :

℞

Iodine pigment,

Creasote,

Oil of cade, āā ʒiv.

Mix.

This he says, in case of early ringworm, is an effectual remedy if well brushed into the roots of the hair. The addition of a quantity of iodine makes the preparation more valuable.

The *iodine pigment* of the British writers is made by dissolving one drachm of iodine in one ounce of alcohol, and allowing the solution to stand in a glass-stopped bottle for several months before it is used, when it will become thick and syrupy.

**Facial Neuralgia.**

A critical synopsis of the results of the various methods of treatment of facial neuralgia is given in Schmidt's Jahrbucher, B. 184, No. 10, 1879, page 48, by Dr. Deahna, of Stuttgart. A thorough historical and critical investigation of all the recorded cases gives rise to the following results: I. Excision of a piece of the nerve was performed in 232 cases, with a mortality of 3 per cent. and only 3.05 per cent. of permanent cures. II. Ligature of the carotid artery, 9 cases on record, with 5.2 per cent. mortality, sometimes grave brain symptoms, and 10.2 per cent. of permanent recoveries. III. Under electrical treatment, the 73 cases on record give 45 per cent. of permanent relief. IV. Nerve stretching has only been employed in a limited number of cases, but with invariable, immediate and lasting good effect. Consequently, the latter method should be resorted to when treatment by electricity has been tried without success.

---

**Erectile Tumor.**

Dr. A. Pupi, in Lo Sperimentale, narrates a case of erectile tumor treated by parenchymatous injection of chloral. The tumor was in the naso-palpebral region, and had extended rapidly. After considering the various methods possible for its removal. Dr. Pupi decided to adopt injection of chloral, remembering that it coagulates the blood, is a hæmostatic and cicatrisant. Three injections were made at intervals of a fortnight, into the substance of the tumor. The proportion of chloral was ten per cent. in distilled water. Each injection was immediately followed by swelling, which, however, was not painful, and disappeared in four or five days. The cure was complete, and scarcely any traces of tumor can now be seen. —*Medical Press and Circular.*

---

**Puerperal Malarial Fever.**

Dr. Fordyce Baker, in the American Journal of Obstetrics, April, 1880, describes this form of puerperal fever under the following diagnostic signs: In the onset the most prominent symptoms are chills, sometimes very slight, temperatures one to two degrees higher than in the beginning of any other puerperal disease. The pulse is rapid and the prostration is greater than is usual with diseases of this period. The second day marks a remission. In two or three days the attack recurs. This form of malarial fever may be developed at any period following parturition, until the physological changes of puerperal convalescence are completed. The large majority of cases are characterized during three or four days before the explosion by general malaise, pain in the head, back and bones, insomnia, thirst, loss of appetite. The differential diagnosis between this and septicæmia depends upon the occurrence or non-occurrence of a fall of temperature of three or four degrees, and a corresponding decline of other symptoms, and upon the fact that septicæmia is rarely accompanied by pain of the head, back or limbs, but is characterized rather by acuteness than bluntness of the sensibilities. Puerperal fever generally occurs between the first and third days after delivery, and is not marked by recurrent chills or by remissions. It may be confounded with phlebitis. The treatment is that of malarial fever.

---

**Chronic Nervous Exhaustion.**

One of the first things needful to be accomplished by medicine is to diminish reflex excitability of the nervous system, especially of the vaso-motor division of the same. For the comfort of the patient, as well as a means for recovery, it is necessary to put out of sight, as far as can be safely done, undue reflex excitability, especially of the cardiac and vaso-motor nervous apparatus. Until this is done it will be impossible for the patient to rest as quietly as needful.

As to the medicinal agents which may be employed in these cases, I have found nothing that answers the purpose better than the milder bromides, associated with digitalis, in some such combination as the following:

℞

Sodii Bromidi, ʒ vij;

Acid Bromohyd. (Squibb's Strong) ʒ iij;

Tr. Digitalis, ʒ iij;

Bismuth Subnit, ʒ iv;

Syr. Prun. Virg., ℥ vij—M.

S. Shake well before using; one or two teaspoonfuls, before or after meals, in water.

---

**Sneezing.**

Surgeon S. Messenger Bradley writes to the *British Medical Journal:* "During the recent rapid changes of temperature I caught a severe cold in my head, accompanied by almost incessant sneezing. My unfortunate nose gave me no rest. The slightest impact of cold air, or passing from the outside air into a warm room, equally brought on a fit of sneezing. In vain I snuffed camphor and pulsatilla; the light catarrh still triumphed over me. At length I resolved to see what the maintenance of a uniform temperature would do toward diminishing the irritability of my Schneiderian membrane, and accordingly I plugged my nostrils with cotton wool. The effect was instantaneous ; I sneezed no more. Again and again I tested the efficacy of this simple remedy, always with the same result. However near I was to a sneeze, the introduction of the pledgets stopped it at once. Nor was there any inconvenience from their presence, making them sufficiently firm not to tickle, and yet leaving them sufficiently loose to easily breathe through." This is really worth knowing, for incessant sneezing is among the greatest of smaller ills, and it seems only a rational conclusion to hope that in this simple plan we may have the most efficient remedy against one of the most distressing symptoms of hay fever.

---

**Diphtheria.**

Dr. A. Cornilleau, of Angers, states that during a severe epidemic of diphtheria, two years ago, he came across a secret preparation, which was sold under the name of "Grande Remede," and which had a most astonishing effect on curing the disease. Having caused an analysis to be made of this remedy, it was found to contain oxalic acid, potassa, and a trace of tannin. He subsequently caused the following compound to be prepared :

℞

Acidi oxalic puri, gm. 1.5–23 grains.

Infusi Thæ vir., " 120 – 4 fl. ʒ.

Syrupi Aurant, " 30 –1½ fl. ℥.

of which a dessert-spoonful was ordered to be administered every three hours. Besides, every hour a bowl or cup (larger or smaller according to the age of the child) of the following infusion was administered :

Fresh sorrel, 150 gm.– 5 ℥.
Boiling water, 1000 gm.–34 fl. ℥.

In all cases, a decided improvement made its appearance from the third day of the attack ; the false membranes diminished in extent as well as in thickness, and recovery generally took place at the end of the week.—*Petit Monit. de la Medicine.*

---

**Hypodermic Syringe Case.**

Thad S. Up de Graff, M. D., Elmira, N. Y., writes to the *Chicago Medical Journal* as follows:

The hypodermic syringe has become an almost indispensable instrument to the physician, and, since it is so extensively employed, any devise that will improve its working qualities will be of service to the profession.

The instances must be rare where the physician has not been perplexed, not to say vexed, when called upon to administer an anodyne to a suffering patient, to find that the piston of his syringe had become perfectly dry, necessitating the loss of much precious time in restoring it to a working condition. Many and loud have been the complaints that are heard of this provoking difficulty with this useful little instrument; and, having suffered the inconvenience many times myself, I sought to remedy it and fully succeeded, in the little devise pictured below:

I sent my syringe to Geo. Tiemann & Co., New York, with instructions to make a hard rubber case just large enough to receive the instrument without its points. This case is filled with a fluid composed of one part glycerine and two parts water, into which is plunged the syringe (the superfluous fluid escaping from the case as the barrel of the syringe is inserted) and the top securely screwed on. It is now air tight, entirely preventing the evaporation of the moisture from the piston and keeping the syringe at all times ready for use. Should no occasion present for its use for six months or a year, yet, when the top is unscrewed and the syringe removed from its bath, it will be found in perfect working order. The rubber case occupies but a trifle more room than the syringe, and is carried in the usual morocco case with the points and bottle of morphine or other solution.

---

**Hydrobromic Ether.**

The new anæsthetic, has been made the subject of careful study by Dr. R. J. Lewis, and the results of his observations have deen published in the Philadelphia *Medical Times*, January 17, 1880. The agent is a colorless liquid, of peculiar odor, intermediate between chloroform and ether in density and volatility, and its vapor is neither inflammable nor irritating to the air passages. Anæsthesia is usually induced in two or three minutes, and recovery of consciousness is of equal rapidity. The mode of administration is by inhalation from a covered napkin, and the quantity required varies with the necessites of the case between one drachm and eleven drachms. The preliminary muscular excitement is moderate and transitory, and is attended with slight acceleration of pulse and slight increase in vascular tension. Respiration is not affected beyond the characteristics of ordinary ether narcosis. Nausea and vomiting do not occur often. The pupils dilate when complete anæthesia is induced and resume their normal dimensions upon the return of consciousness. They may be taken as the guide.—*Chicago Med. Gaz.*

We have used the anæsthetic referred to, in three instances, with the following results: The first case was that of a women, æt 35, suffering from a supposed intra-uterine fibroid. Anæsthesia was desired for thorough examination and operation. Two drachms was given every 10 minutes until eight drachms were inhaled, causing great excitement of the patient, without inducing insensibility. Chloroform was at last resorted to, to produce the condition desired. It was next given to a child æt 6, for an operation for strabismus. Two drachms produced complete anæsthesia in five minutes, lasting for ten minutes, when the patient became suddenly conscious and walked away without suffering the least inconvenience. The next administration was to an adult for an iridectomy. Six drachms were used producing complete insensibility in 20 minutes. There was no rigidity of the muscles, no nausea, no stertorious breathing, no failing of the pulse. We are inclined to look upon it as a safe anæsthetic,—better than ether, not so satisfactory as chloroform, and not a whit less dangerous, where the latter is skillfully administered.—[ED. BISTOURY.]

---

**Night-Sweating of the Phthisis.**

Dr. William Murrell has recently made, at Dr. Ringer's suggestion, some observations on the influence of nitrite of amyl on the night-sweating of phthisis. The patients were seventeen in number, all adults—thirteen men and four women. All stages of the disease were represented; in some cases there was considerable elevation of temperature, whilst in others the lung mischief was latent. The majority of the patients were seen daily for some weeks, and some were under observation for three months. The medicine was given internally at bed-time, the dose varying from a half to three minims. For convenience in dispensing, a one in ten solution in rectified spirit was usually employed, but in some cases the amyl was given in suspension in water or on sugar.

In three out of the seventeen cases no benefit was experinced from the treatment. These patients were all men. One had suffered from profuse perspiration all his life, not only at night, but also in the day-time, and he was covered with moisture on the slightest exertion even in the dead of winter. The amyl was given nightly in minim doses for a fortnight without checking the perspiration in the slightest degree. He had previously been treated unsuccessfully with oxide of zinc, hypodermic injections of atropia, and drugs. On one occasion he was freely rubbed all over with belladonna liniment till his pupils were fully dilated, but the sweating continued as before. The second was a case of advanced phthisis, in which the amyl was given nightly for a fortnight, in doses varying from one to three minims, without benefit; oxide of zinc subsequently failed. In the third unsuccessful case the patient had hemiplegia and tertiary syphilis, in addition to his lung mischief. The amyl was taken in drop doses for eight nights, and seemed rather to increase than to diminish the amount of perspiration; in this case, too, oxide of zinc was given without benefit.

In the remaining fourteen cases the treatment was successful. The most striking case was that of a young man who had suffered severely from night-sweating for six weeks. A single dose of the amyl stopped them at once and completely for a fortnight. The perspirations then returned, and a single dose again kept them in check for a fortnight. For the third time this was tried and with like result. It may have been a mere coincidence, but it certainly appeared to be the result of the treatment. In the majority of cases the treatment was less successful. Usually on the first night little or no benefit was experienced; on the next night the perspiration was less, and it gradually decreased in severity night by night till, at the expiration of a fortnight, it had nearly, if not wholly ceased, and the patient was able to discontinue the medicine. At the expiration of about a week the perspiration would return, and it would be necessary to give the medicine again. One of the patients had renal disease in addition to the lung mischief, and another had frequent hæmoptyisis. The others were simple cases of phthisis. Most of them were able to take out-door exercise, but two or three were confined to bed.

Nitrite of amyl is a good remedy for night-sweats, but for promptness of action is decidedly inferior to atropia and other remedies.—*Medical News*, from *Practitioner*.

## Figures and Tables

**Figure f1:**